# Role for colorectal teams to support non-colorectal teams to improve clinical outcomes and adherence to ERAS guidelines for segmental colectomy: a cohort study

**DOI:** 10.1186/s12893-021-01149-8

**Published:** 2021-03-16

**Authors:** Simonette R. Mallard, Kari A. Clifford, R. Park, Trainee Intern Research Group, Annie Chan, Aqilah Hashim, Wafaatiqah Jufri, Zeong Kang, Terrence Reilly, Cara Woodhead, Holly McLaren, Kim Cousins, Ann Patton, John C. Woodfield, Mark Thompson-Fawcett

**Affiliations:** 1 Department of Surgical Sciences, Dunedin Medical Campus, Otago Medical School, University of Otago, PO Box 56, Dunedin, 9054 New Zealand; 2 Department of Preventive and Social Medicine, Dunedin Medical Campus, Otago Medical School, University of Otago, Dunedin, New Zealand; 3 Dunedin Public Hospital, Dunedin, New Zealand

**Keywords:** ERAS, Colorectal surgery, Laparoscopy, Length of stay, Segmental colectomy

## Abstract

**Background:**

To identify whether compliance with Enhanced Recovery After Surgery (ERAS) Society recommendations is associated with length of stay (LOS) in a New Zealand hospital for patients undergoing segmental colectomy in mixed acute and elective general surgery wards.

**Methods:**

Consecutive elective colorectal surgeries (n = 770) between October 2012 and February 2019 were audited. Patients with non-segmental colectomies, multi-organ surgeries, LOS > 14 days, and those who died were excluded. Logistic regression was used to determine the relationship between patient demographics, compliance with ERAS guidelines, and suboptimal LOS (> 4 days).

**Results:**

Analysis included 376 patients. Age, surgery prior to 2014, surgical approach, non-colorectal surgical team, operation type, and complications were significantly associated with suboptimal LOS. Non-compliance with ERAS recommendations for laparoscopy [OR 8.9, 95% CI (4.52, 19.67)], removal of indwelling catheters (IDC) [OR 3.14, 95% CI (1.85, 5.51)], use of abdominal drains [OR 4.27, 95% CI (0.99, 18.35)], and removal of PCA [OR 8.71, 95% CI (1.78, 157.27)], were associated with suboptimal LOS (univariable analysis). Multivariable analysis showed that age, surgical team, late removal of IDC, and open approach were independent predictors of suboptimal LOS.

**Conclusions:**

Non-compliance with ERAS guidelines for laparoscopic approach and early removal of IDC was higher among procedures performed by non-colorectal surgery teams, and was also associated with adverse postoperative events and suboptimal LOS. This study demonstrates the importance of the surgical team’s expertise in affecting surgical outcomes, and did not find significant independent associations between most individual ERAS guidelines and suboptimal LOS once adjusting for other factors.

## Introduction

Colorectal surgery is associated with a long length of stay (LOS) [1], contributing significantly to the cost of healthcare [2]. In order to address this, the Enhanced Recovery After Surgery (ERAS) Society developed 20 recommendations, aiming to reduce morbidity and LOS following elective colorectal surgery [3]. In comparison with traditional care, prospective studies [4, 5] and randomized controlled trials [6] have demonstrated a reduction in morbidity and LOS in hospital following implementation of ERAS protocols.

While overall adherence to an ERAS protocol results in improved postoperative outcomes, the importance of individual recommendations is less clear [7]. Recommendations found to be independently associated with LOS include avoidance of intraoperative drain placement [8–10], avoidance of nasogastric intubation [8–12], early removal of indwelling catheters (IDC) [8–10, 12], and early postoperative mobilisation [9–12]. A laparoscopic, rather than an open, approach is also shown to result in earlier discharge from hospital [9, 11, 13, 14]. However, lack of clear evidence supporting individual ERAS recommendation has led hospitals to tailor protocols to their own settings.

Use of an ERAS protocol for colorectal surgery in Dunedin Public Hospital began in 2012, and was associated with a decrease in median LOS from 9 to 7 days. In our context, ERAS patients are not physically segregated, or cared for by dedicated ERAS nurses. However, LOS after segmental colectomy has remained suboptimal; below the generally accepted median of 4–5 days [2, 10, 15]. Therefore, the objective of our study was to investigate compliance with ERAS Society recommendations in Dunedin Public Hospital, and whether compliance was related to suboptimal LOS.

## Methods

A clinical audit was performed on 770 consecutive cases of patients undergoing elective colorectal surgery at Dunedin Public Hospital (Dunedin, New Zealand) between October 2012 and February 2019. ERAS patients are cared for by a 0.5 full-time equivalent nurse in an acute and elective general surgery ward and are not segregated. Segmental colectomies are performed by colorectal and non-colorectal teams. A colorectal team was defined as a team lead by two or three consultants who had performed sub speciality colorectal training and where the consultants’ elective workload was primarily colorectal surgery. The ERAS database contains prospectively recorded details including patient and surgical-procedure related characteristics, and compliance with protocol items.

Patients who underwent elective segmental colectomies within the study period were included. Those receiving low anterior resections and undergoing multi-organ surgeries were excluded. On the basis that significant complications were unlikely to be due to non-compliance with ERAS, but likely to extend stay and potentially confound analyses, cases with greater than 14 days stay were excluded. Patient deaths within 14 days were excluded from analyses.

A qualitative investigation using clinical notes was undertaken to explore factors delaying discharge. Clinical notes of 10 consecutive segmental colectomy patients recorded in the ERAS database were reviewed by two researchers, and factors delaying discharge were recorded.

### Statistical analysis

Suboptimal LOS in hospital was defined as > 4 days [15, 16] and LOS was dichotomized into optimal or suboptimal. Logistic regression analyses were performed to estimate unadjusted odds ratios (OR) and 95% confidence intervals (CI) for LOS, with the following variables defined a priori: age (years); sex (male, female); time period of surgery, indicating when two additional colorectal surgeons were employed (2012–2013, 2014–2019); American Society of Anesthesiologists (ASA) score (1–4); surgical team (colorectal, non-colorectal); stoma formed (yes, no); anastomosis formed (yes, no); operation type; length of time between preoperative counselling to surgery (days); and non-compliance with ERAS recommendations. Significant predictors in simple logistic regression were evaluated for correlation, with Cramer’s V calculated for correlations between categorical variables. Covariates were limited to a minimum of 10 events per response variable [17]. Stepwise multiple logistic regression analysis was undertaken with these significant predictors, and generalised variance inflation factors (VIF) were calculated to determine multicollinearity among the factors. Goodness of fit was assessed with the Hosmer and Lemeshow goodness of fit test and area under the receiver operator curve (AUROC). All analyses were performed using R 4.0.0 [18]. This manuscript complies with the Strengthening the Reporting of Observational Studies in Epidemiology (STROBE) guidelines for reporting observational studies [19].

## Results

**Fig. 1.**
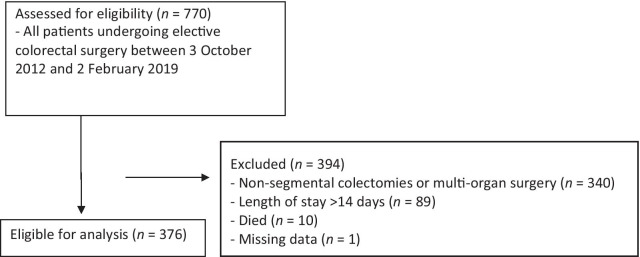
CONSORT flow diagram of patient eligibility. Some patients met multiple exclusion criteria

Between the 3rd of October 2012 and the 2nd February 2019, 770 patients underwent colorectal surgery on the ERAS pathway at Dunedin Hospital. We excluded 394 patients for the following: non-segmental colectomy or multi-organ surgery (340), LOS > 14 days (89), and death during admission (10) (Fig. 1).

Some patients met multiple exclusion criteria. Of the 43 patients who were excluded solely on the basis of having a LOS of > 14 days, 39 experienced postoperative complications. These included prolonged ileus (28), anastomotic leak (10), small bowel obstruction (1), aspiration pneumonia (1), *C. difficile* infection (1), and ischaemic bowel involving stoma (1). Some patients had multiple complications.

Patient demographics and surgical procedure-related characteristics are presented in Table 1.

**Table 1:** Patient and surgical procedure-related characteristics

Characteristic	Overall	LOS ≤ 4 days	LOS > 4 days
Total sample, *n* (%)	376	88 (23)	288 (77)
Median LOS, days (IQR)	6 (5–8)	4 (4–4)	7 (6–9)
Age, median years (IQR)	74.5 (66–80)	71 (42–79)	75.1 (67–81)
Days between counseling and surgery, median (IQR)	8 (3–8)	8 (5–8)	8 (3–8)

	*n* (%)	*n* (%)	*n* (%)
Sex			
Female	189 (50)	45 (51)	144 (50)
Male	187 (50)	43 (49)	144 (50)
Year of surgery			
2012 to 2013	83 (22)	6 (7)	77 (27)
2014 to 2016	293 (78)	82 (93)	211 (73)
ASA category			
1	20 (5)	7 (8)	13 (5)
2	171 (46)	47 (53)	124 (43)
3	135 (36)	25 (28)	110 (38)
4	9 (2)	1 (1)	8 (3)
Not recorded	41 (11)	8 (9)	33 (12)
Surgical approach			
Laparoscopic	222 (59)	79 (90)	143 (50)
Open	146 (39)	8 (9)	138 (48)
Conversion	8 (2)	1 (1)	7 (2)
Type of surgery			
Right hemi-colectomy	196 (52)	44 (50)	152 (53)
Left hemi-colectomy	19 (5)	7 (8)	12(4)
High anterior resection	97 (26)	34 (39)	63 (22)
Sigmoid colectomy	33 (9)	1 (1)	32 (11)
Transverse colectomy	13 (4)	2 (2)	11 (4)
Subtotal colectomy	18 (5)	0	18 (6)
Surgical team			
Colorectal team	223 (59)	78 (89)	145 (50)
Non-colorectal team	153 (40)	10 (11)	143 (50)
Stoma formed			
No	359 (96)	86 (98)	273 (95)
Yes	17 (5)	2 (2)	15 (5)
Anastomosis formed			
No	15 (4)	2 (2)	13 (5)
Yes	361 (96)	86 (98)	275 (96)
Complications			
No	273 (73)	85 (97)	188 (65)
Yes	99 (26)	2 (2)	97 (34)
Not recorded	4 (1)	1 (1)	3 (1)

*ASA* American Society of Anesthesiologists, *IQR* interquartile range, *LOS* length of stay

Seventy-six percent of patients stayed in hospital > 4 days, with a median LOS of 7 days (interquartile range [IQR] 6 to 9 days). Of patients staying ≤ 4 days, the majority underwent laparoscopic segmental colectomies (89%) and were operated on by colorectal surgeons (89%).

Following the increase in the proportion of cases done by laparoscopic surgery in 2014, the proportion of patients with a LOS > 4 days was reduced from 93 to 72%, and the median LOS fell from 8 (IQR 7, 10) days to 6 (IQR 4, 8 days). In addition, the proportion of surgeries performed laparoscopically rose from 7 to 74%, and readmission within 30 days fell from 10 to 6%.

Fifteen of the 20 ERAS Society recommendations are recorded in the database, and compliance was close to 100% for the following (Table 2): preadmission education, preoperative bowel preparation, postoperative nausea and vomiting prevention, intraoperative hypothermia prevention, and a step down regime or pain team utilized for postoperative analgesia management.

**Table 2:** Compliance with Enhanced Recovery After Surgery (ERAS) Society recommendations recorded in the electronic ERAS database (*n* = 376)

ERAS Society recommendation		% Compliance	
	Overall *n* (%)	LOS ≤ 4 days *n* (%)	LOS > 4 days *n* (%)
Preadmission education	376 (100)	88 (100)	288 (100)
No preoperative bowel preparation	370 (98)	87 (99)	283 (98)
Preoperative fluid loading	245 (65)	96 (65)	149(65)
Postoperative nausea and vomiting prevention			
Intraoperative	303 (81)	75 (85)	228 (79)
Postoperative	369 (98)	87 (99)	282 (98)
Laparoscopic approach	222 (59)	79 (90)	143 (50)
No nasogastric intubation	373 (99)	88 (100)	285(99)
Abdominal drains	350 (93)	86 (98)	262 (91)
Restricted perioperative fluid management	131 (35)	30 (34)	101 (35)
Intraoperative hypothermia prevention	369 (98)	87 (90)	282 (98)
Indwelling catheter removed on POD 1	210 (56)	67 (76)	143 (50)
PCA removed on POD 2	292 (78)	71 (81)	221 (77)
Step down regime or pain team used	374 (99)	87 (99)	287 (99)
Nutritional supplement provided	254 (68)	65 (74)	189 (66)
First standard meal consumed on DOS	71 (19)	13 (15)	58 (20)
Early mobilisation within 24 h	209 (56)	51 (58)	158 (55)

*DOS* day of surgery, *LOS* length of stay, *PCA* patient controlled analgesia, *POD* postoperative day

Less than 50% compliance was achieved for restricted perioperative fluid management (35%), and first standard meal consumed on the day of surgery (29%).

Logistic regression results of patient demographics, surgical procedure-related characteristics, and compliance with ERAS guidelines in relation to suboptimal LOS are shown in Table 3 for items with less than 100% compliance.

**Table 3:** Logistic regression analysis for factors associated with suboptimal length of stay

	Univariable analysis			Multivariable analysis		
	Odds ratio	95% CI	*P*	Odds ratio	95% CI	*P*
Age	1.02	(1.02, 1.06)	0.022*	1.02	(0.99, 1.05)	0.054*
Sex (Male)	1.04	(0.65, 1.69)	0.852			
Pre 2014	4.98	(2.26, 13.22)	< 0.001***			
ASA fitness grade						
II	1.86	(0.76, 4.94)	Wald 0.15			
III	1.42	(0.51, 3.69)				
IV	4.30	(0.59, 88.43)				
Surgical team (non-colorectal)	7.69	(3.99, 16.37)	< 0.001***	4.09	(1.85, 9.05)	< 0.001***
Stoma formed	2.36	(0.64, 15.18)	0.260			
Anastomosis formed	0.49	(0.075, 1.82)	0.356			
Complications	21.93	(6.72, 134.9)	< 0.001***			
Days between counselling and surgery	1.00	(0.98, 1.03)	0.741			
Operation			Wald < 0.007**			0.34
Left hemicolectomy	1.81	(1.09, 3.17)		0.59	(0.18,1.91)	
Right hemicolectomy	1.86	(1.09, 3.17)		1.17	(0.64, 2.15)	
Sigmoid colectomy	11.77	(2.89, 107.88)		7.75	(0.88, 68.18)	
Subtotal colectomy	20.10	(2.59, 2588.14)		> 100	(0, Inf)	
Transverse colectomy	2.49	(0.68, 13.42)		2.3	(0.41, 12.6)	

Non-compliance with ERAS recommendations						
Preoperative bowel prep avoided	0.65	(0.03, 4.10)	0.697			
Preoperative fluid loading	1.04	(0.64, 1.74)	0.870			
Intraoperative PONV prevention	1.52	(0.81, 3.02)	0.211			
Postoperative PONV prevention	1.85	(0.31, 32.21)	0.571			
Laparoscopic approach	8.90	(4.30, 18.40)	< 0.001***	2.92	(1.26, 6.78)	0.012 **
NG intubation	> 100	(0, Inf)	0.986			
Abdominal drains avoided	4.27	(0.99, 18.35)	0.051*	2.31	(0.42, 12.6)	0.307
Restricted perioperative fluid management	0.96	(0.57, 1.57)	0.866			
Intraoperative hypothermia prevention	1.85	0.31, 35.21)	0.571			
Indwelling catheter removed on POD1	3.14	(1.85, 5.51)	< 0.001***	1.93	(1.04, 3.59)	0.035*
PCA removed on POD2	8.71	(1.78, 157.27)	0.036*			
Nutritional supplement provided	1.48	(0.87, 2.56)	0.150			
First standard meal consumed on DOS	0.75	(0.36, 1.60)	0.44			
Early mobilisation within 24 h	1.13	(0.70, 1.85)	0.609			
Stepdown pain relief	3.29	(0.13, 84.00)	0.400			

Odds ratio for age and days between counselling and surgery is the change in odds of LOS > 4 days for each unit change of the variable. For categorical variables, it is the change in odds of LOS > 4 compared to the reference category, in the case of ERAS recommendations, compliance is the reference. 95% CI is defined as the 95% confidence interval for the estimated odds ratio

*PONV* postoperative nausea and vomiting, *NG* nasogastric, *PCA* patient controlled analgesia, *DOS* day of surgery, *POD* postoperative day, *LOS* length of stay

*Significant p ≤ 0.05

**Significant p ≤ 0.01

***Significant p ≤ 0.001

Surgeries performed between 2012 and 2013 were more likely to result in LOS > 4 days than those performed between 2014 and 2019 [OR 4.98, 95% CI (2.26, 13.22); *P* < 0.001]. In addition, the odds of a suboptimal LOS were significantly greater when the surgery was performed by a non-colorectal team [OR 7.69, 95% CI (3.99, 16.37); *P* < 0.001], and when a patient developed a postoperative complication [OR 21.93, 95% CI (6.72, 134.90); *P* < 0.001].

An open surgical approach was shown to significantly increase the odds of a suboptimal LOS [OR 8.90, 95% CI (4.52, 19.67); *P* < 0.001]. The odds of a suboptimal LOS were increased for failing to remove an indwelling catheter on postoperative day 1 [OR (95% CI) 3.14, (1.85, 5.51); *P* = 0.001], patient controlled analgesia (PCA) on day 2 [OR (95% CI) 8.71 (1.78, 157.27); *P* = 0.036], or use of abdominal drains [OR (95% CI) 4.27 (0.99, 17.60); *P* = 0.036]. Compliance with other ERAS Society recommendations was not statistically significantly associated with a suboptimal LOS.

Age, surgical team, operation, laparoscopic approach, use of abdominal drains, and IDC removal were included in the final logistic regression model (Table 3). This model was reliable (Hosmer–Lemeshow *P* = 0.840) and accurate (AUROC (or C-index) = 0.809). There was no evidence of multicollinearity, all VIF ≤ 2.0.

The multivariable analysis showed that a non-colorectal surgical team [adjusted OR (95% CI)] [4.09 (1.76, 10.5)], non-laparoscopic approach [2.92 (1.26, 6.78)], and failure to remove IDC on postoperative day 1 [1.93 (1.04, 3.59)] increased the likelihood of suboptimal LOS in this model. Use of abdominal drains and operation were not independently associated with suboptimal LOS in the multivariable model. Sensitivity analyses including patients with a LOS of more than 14 days yielded similar results.

Qualitative investigation using the clinical notes of ten segmental colectomy patients recorded in the ERAS database found that discharge was delayed in some cases when discharge criteria were met during the weekend. The need for transfer to a distant geographical area and psychosocial concerns delayed weekend discharges in several cases.

## Discussion

In this study of 376 patients undergoing elective segmental colectomies at a tertiary New Zealand Hospital, only 23% met the target LOS (≤ 4 days). We identified several factors that were associated with a suboptimal LOS. These included age, complications, laparoscopy, and procedures performed by non-colorectal teams. Among ERAS recommendations, only open approach and failure to remove IDC on postoperative day 1 were independently associated with suboptimal LOS, which is similar to the findings of other studies investigating ERAS compliance [20]. Of these ERAS recommendations, overall compliance was 59% for a laparoscopic approach and 56% for IDC removal, with lower compliance among non-colorectal teams (27% and 47%, respectively). These findings indicate that increased adherence to these recommendations, particularly by non-colorectal teams, may reduce hospital stay in this setting.

With respect to uptake of laparoscopy, changes over the time period studied suggests that this directly impacted the LOS. The hiring of two additional colorectal surgeons in 2014 was followed by an increase in compliance with the ERAS recommendation for a laparoscopic approach (from 7 to 74% of segmental colectomies) with a concomitant reduction in the median LOS from 8 to 6 days. However, in multivariable analysis, not taking a laparoscopic approach was significantly associated with increased likelihood of a suboptimal LOS, independently of the surgical team.

Limited knowledge of discharge criteria for ERAS patients among junior staff, and a lack of clear discharge planning are also potentially responsible for delays in discharge over weekends. Contributing factors in other delayed discharges included psychosocial concerns, such as patient expectations and lack of support at home.

The strong association between a laparoscopic approach and reduced LOS has also been noted in other studies [9, 11, 13, 14, 20]. It has been proposed that the reduction in surgical trauma, postoperative pain, and ileus, as well as a smaller surgical incision, are all reasons why patients undergoing laparoscopic surgery are discharged earlier [23]. However, colorectal teams were more compliant than non-colorectal teams with some protocol items, including laparoscopic approach (81% vs 27%) and IDC removal (65% vs 34%), which may have been due to greater experience with the colorectal ERAS pathway. This includes colorectal surgeons being more confident to discharge patients as soon as they met specified discharge criteria. In a hospital context with mixed acute and elective general surgery practice, we would suggest colorectal teams support non-colorectal teams in implementing an ERAS pathway for their colorectal patients, rather than suggesting all segmental colectomies should be done by a colorectal subspecialist.

The prevalence of complications was significantly greater among patients without timely removal of IDC and PCA, and use of abdominal drains, when compared with patients whose care adhered to guidelines (49%, 47%, and 55%, respectively, versus 26% overall; all *P* ≤ 0.001). While some complications, such as wound infection and anastomotic leak, will not be related to IDC, abdominal drains, and PCA; others such as ileus and urinary tract infections may be. These ERAS items have been previously identified as predictors of increased LOS if not followed [12]. They were significant predictors in the univariable analysis, and IDC removal was independently associated with LOS in the multivariable analysis. However, abdominal drain removal and discontinuation of PCA on postoperative day two were not independent predictors of suboptimal LOS in multivariable analysis. This indicates that the effects of abdominal drain or PCA removal on suboptimal LOS could be explained by the association with postoperative complications and surgical team.

Our analysis found an association between age and LOS in the multivariable model. Previous research has shown an increasing risk of suboptimal LOS with increasing age [9, 12], and that younger patients are more likely to have a reduced LOS [15, 24]. An increased ASA classification has also been shown to be associated with LOS [9, 12, 14]. Age and ASA were highly correlated in our study, and while there was no indication of multicollinearity, patient age was a better predictor of suboptimal LOS than ASA.

A major strength of our study was the use of data recorded prospectively, rather than retrospectively from clinical notes that may be subject to bias. Information was also recorded in the database by a single collector, thereby preventing inter-observer variability. This study may be limited by the application of minimal exclusion criteria, an elderly patient-base, and the lack of a specifically designated ERAS area on the ward, or limited specialised ERAS nursing staff. Nonetheless, our LOS of 6 days from 2014 onwards was comparable to other similarly-sized studies [23–25]. Overall, we had a longer LOS than observed in similar populations in New Zealand [26], and potential reasons for this were identified in our qualitative review of the clinical notes, including delays in discharge related to residence in distant geographical areas, lack of support at home, and delays in discharge during weekends. We believe further investigation of these factors is warranted.

## Conclusions

A reduction in LOS was observed following the implementation of an ERAS protocol for elective colorectal surgery in Dunedin Public Hospital, with a further reduction in 2014 when two additional colorectal surgeons were employed, enabling more laparoscopic surgery. After adjusting for patient demographics, operation type, and surgical team, potentially modifiable factors related to LOS were IDC removal on postoperative day 1, and laparoscopic approach. Concentrating on supporting non-colorectal teams in using the ERAS pathway, patient and clinician education, and addressing issues surrounding the management of patient discharge, may optimise LOS after segmental colectomy.

## Data Availability

The datasets used and analysed during the current study are available from the corresponding author on reasonable request.

## Abbreviations

ASA: American Society of Anaesthesiologists
AUROC: Area under the receiver operator curve
DOS: Day of surgery
ERAS: Enhanced Recovery After Surgery
IDC: Indwelling catheter
LOS: Length of stay
OR: Odds ratio
NG: Nasogastric
PCA: Patient controlled analgesia
PONV: Postoperative nausea and vomiting
POD: Post operative day
STROBE: Strengthening the Reporting of Observational Studies in Epidemiology
VIF: Varience inflation factor
